# The Six-Legged Subject: A Survey of Secondary Science Teachers’ Incorporation of Insects into U.S. Life Science Instruction

**DOI:** 10.3390/insects9010032

**Published:** 2018-03-14

**Authors:** Erin Ingram, Douglas Golick

**Affiliations:** Department of Entomology, University of Nebraska‒Lincoln, Lincoln, NE 68588, USA; dgolick2@unl.edu

**Keywords:** entomology education, biology, life science, in-service teachers, insect, arthropod

## Abstract

To improve students’ understanding and appreciation of insects, entomology education efforts have supported insect incorporation in formal education settings. While several studies have explored student ideas about insects and the incorporation of insects in elementary and middle school classrooms, the topic of how and why insects are incorporated in secondary science classrooms remains relatively unexplored. Using survey research methods, this study addresses the gap in the literature by (1) describing in-service secondary science teachers’ incorporation of insects in science classrooms; (2) identifying factors that support or deter insect incorporation and (3) identifying teachers’ preferred resources to support future entomology education efforts. Findings indicate that our sample of U.S. secondary science teachers commonly incorporate various insects in their classrooms, but that incorporation is infrequent throughout the academic year. Insect-related lesson plans are commonly used and often self-created to meet teachers’ need for standards-aligned curriculum materials. Obstacles to insect incorporation include a perceived lack of alignment of insect education materials to state or national science standards and a lack of time and professional training to teach about insects. Recommendations are provided for entomology and science education organizations to support teachers in overcoming these obstacles.

## 1. Introduction

With more than 900,000 insect species currently described [1], insects represent more than 50% of all animal life on Earth [2]. In addition to their staggering diversity, insect biomass is equally impressive with recent estimates indicating that for every human on Earth, there are 200 million insects [1]. With insects living practically everywhere, they have been called the most dominant group of animals on Earth [2]. Yet, despite the complexity and evolutionary success of insects, public understanding and appreciation of insects and other invertebrates remains limited [3,4] and views of insects can often be simplified into “beautiful” or “bothersome” [3]. To address this lack of understanding and appreciation for insects and their relatives, entomology education efforts have focused on identifying necessary entomology competencies [5] and providing formal and informal educators with appropriate teaching resources and professional development opportunities [6,7,8].

While most entomologists would likely agree that supporting students’ entomological literacy is a noble goal unto itself, teaching with insects and insect-related materials can also serve broader science education goals. First, science education reform efforts have called for inquiry-based approaches to teaching and learning in the science classroom [9,10]. Inquiry-based science learning promotes student engagement in “the wide range of approaches that are used to investigate, model, and explain the world” [10] (p. 42). Live insects can be used to support inquiry-based approaches via laboratory investigations due to their small size, fast reproductive rate, low cost, easy handling, and few associated research restrictions [6]. To assist teachers in using live insects for classroom investigations, lesson plans and practitioner articles are available to support exploration of key science concepts including growth and development [11,12], evolution and adaptation [13,14,15], structure and function [16,17], behavior [18,19,20,21], and ecology [22,23,24,25,26,27].

Indeed, some studies suggest improved student outcomes result from student investigations or experiences with live insects during instruction. Shepardson [28,29] noted that elementary students’ ideas about insects were likely influenced by formal science experiences including examining butterfly and beetle life cycles in grade 1, raising and feeding butterflies in grade 3, and classifying organisms based on characteristics in grade 5. A review by Killerman [30] indicated that having experiences with living invertebrates including insects had a more positive impact on students’ attitudes and academic performance when compared to a control group which received no instruction and another experimental group which studied the same animals with pictures, slides, models, and preserved animals. Sammet and Dreesmann [31] reported greater knowledge acquisition for students who participated in direct observation, hands-on investigation, and care for an ant colony than students in a control group lacking these experiences.

In addition to supporting students’ cognitive gains in science, insects may provide opportunities to improve student attitudes toward science. While young students are reported to have positive science experiences, research has shown that student interest and motivation in school science declines throughout secondary grade levels [32,33]. This trend continues as approximately 30% of U.S. undergraduate students intend to major in science, technology, engineering, or mathematics (STEM) disciplines, but only about half of these students go on to graduate with a STEM degree [34] (p. 98). Interviews with early secondary science students have found that declining attitudes toward school science are due, in part, to a perceived lack of relevance to daily life and a lack of hands-on experiences [35]. As insects impact our daily lives as model organisms in medicine and basic research, decomposers, pollinators, agricultural pests, and vectors of human and other animal diseases [2], they are ideal organisms for exploring the everyday relevance of science. In addition, the wide variety of inquiry-based insect investigations previously mentioned also potentially mitigate student concerns about the lack of hands-on experiences in the life science classroom.

Lastly, recent transformations in science education have called for “raising engineering design to the same level as scientific inquiry in science classroom instruction at all grade levels” [10] (p. xiii). With increased emphasis placed on engineering, there is increased demand for K-12 teaching resources that allow students to examine real-world problems and engineer potential solutions. To meet this need, some insect-related teaching resources [36,37] have been developed in which students can examine pollinator and other biodiversity losses and engineer man-made nesting domiciles or other conservation solutions to aid in insect conservation efforts. By supporting engineering design in K-12 classrooms through insect-related curriculum, not only can science education goals be addressed, but efforts from these projects can also work in concert with the entomology community in addressing authentic local and global issues.

To better understand the potential benefits of including insects and insect-related content in formal science education settings, it is important to explore how entomology education currently exists in science classrooms. A review of the literature suggests that the process of how and why insects are used in secondary science classrooms (grades 9–12) remains relatively unexplored. Using survey research methods, this study seeks to address the gap in the literature by (1) describing the prevalence and nature of insect incorporation in secondary life science instruction; (2) identifying factors that support or deter the incorporation of insects into science instruction; and (3) understanding how the science education and entomology communities can support teachers in their entomology education efforts. Findings from this study will provide insight into content and instructional practices of in-service (currently employed) secondary science teachers and can be used to develop appropriate resources to address potential gaps in curriculum offerings and student understanding.

## 2. Materials and Methods

### 2.1. Survey Development

The survey was constructed using the tailored design method [38] and distributed via Qualtrics Survey Software (Provo, UT, USA). Our instrument was limited to a maximum of 24 questions to minimize participant dropout due to fatigue. The majority of participants (73%) required 10–20 min to complete the survey. Both closed-ended and open-ended questions were included to collect quantitative and qualitative data. The instrument included 1 item to confirm participants were consenting to participate in a research survey. Remaining items allowed for collection of data on the following:

- Teacher demographics (8 items)
- Description of insect incorporation such as frequency or type of insect used (9 items)
- Barriers to incorporation (1 item)
- Teacher attitudes (2 items)
- Preferred resources to improve future incorporation (2 items)
- Perceived student benefit (1 item).

To maximize content validity, four high school biology teachers with appropriate content knowledge and/or experience related to the construct of “insect incorporation” piloted the survey for clarity and content prior to implementation.

### 2.2. Participant Recruitment

We licensed 2000 high school life science teacher email addresses from MCH Strategic Data (Silver Springs, MO, USA), a compiler of national education data. An initial recruitment email was distributed to 2000 potential participants over a three-day period. Two follow-up reminder emails were sent to all potential participants who had not yet taken part in the survey. The first reminder was sent one week after the initial email and the second follow-up was sent a week later.

All subjects gave their informed consent for inclusion before they participated in the study. The study was conducted in accordance with the Declaration of Helsinki, and the protocol was approved on 1 April 2015 by the Ethics Committee of University of Nebraska–Lincoln institutional review board (IRB#20150415217 EX).

### 2.3. Data Analysis

Quantitative data were analyzed using IBM SPSS Statistics for Macintosh (Version 22; IBM Corp., Armonk, NY, USA). Descriptive statistics including frequency, rank, and mean were calculated to quantitatively describe the process of insect incorporation.

## 3. Results

### 3.1. Accuracy and Representativeness of Sample

Survey data were collected from 254 high school life science teachers resulting in a 12.7% response rate. Our survey data set includes teachers from 41 different states in the U.S. The top six states represented in the survey were California, Texas, Maryland, Pennsylvania, Wisconsin, and New York. Based on an estimated 59,000 total high school biology teachers in the U.S. [39], our reported percentage estimates have a margin of error of 6.1% at the 95% confidence interval based on a standard calculation for margin of error [38].

To determine if our sample was representative of the population of inference we compared participant demographics to U.S. natural science teacher demographics from the U.S. Department of Education’s National Center for Education Statistics [40]. Based on these comparisons (Table 1), our sample tended to be slightly older (18% more than average are 40 years or older), more female (7% more than average), with more teaching experience (22% more than average with 10 or more years of teaching experience), and with a higher degree (15% more than average with a masters or doctorate degree). Our sample was over-representative of suburban schools (9% more than average) while slightly under representing those schools in cities and rural areas (−3% and −6%, respectively). In addition public schools were slightly overrepresented in our survey sample (+12%) compared to private schools when compared to national statistics [41].

### 3.2. Limitations

We recognize that entomology could be considered a topic of specific interest for a limited number of secondary science teachers and that this specificity could result in self-selection bias in our survey sample. This may prevent the generalization of these results to describe the prevalence of insect-related materials in U.S. secondary life science classrooms as a whole. However, we have provided data to indicate how well our survey sample aligns with known parameters for national teacher and school demographics. Using these comparisons as evidence, the survey does provide information about insect incorporation practices in a representative sample of U.S. secondary life science classrooms.

### 3.3. Description of Insect Incorporation in High School Biology Classrooms

**Figure 1:**
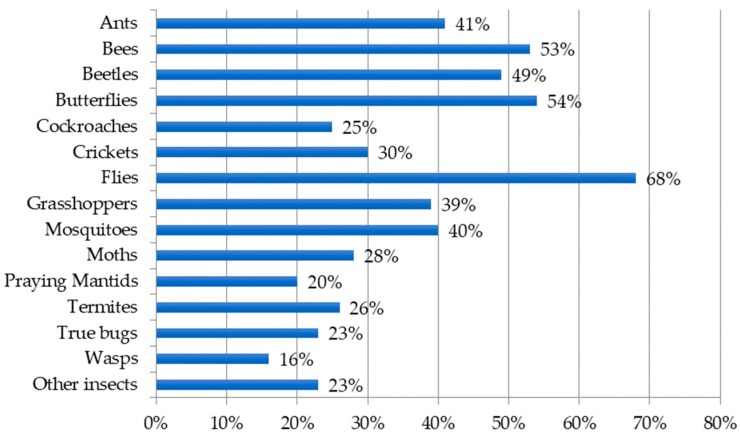
Insect types incorporated.

Most teachers (88%) responding to the survey incorporated insects to some degree within a typical academic year. Within the survey, insect incorporation was defined as “presentation of or interaction with any media depicting an insect such as a picture, video, audio, text, lecture, discussion, activity, lesson, pinned specimen, or live insect.” Teachers used a wide diversity of insect types (Figure 1) with an average of 5.27 ± 0.26 SE insect types used in their instruction. Of the 48 teachers who reported incorporating “other insects”, popular responses included reference to other arthropods (*n* = 16) including isopods, centipedes, millipedes, mites, or ticks; aquatic insects (*n* = 12) such as caddisflies, mayflies, dragon- or damselflies, or stoneflies; and walking sticks (*n* = 6).

**Figure 2:**
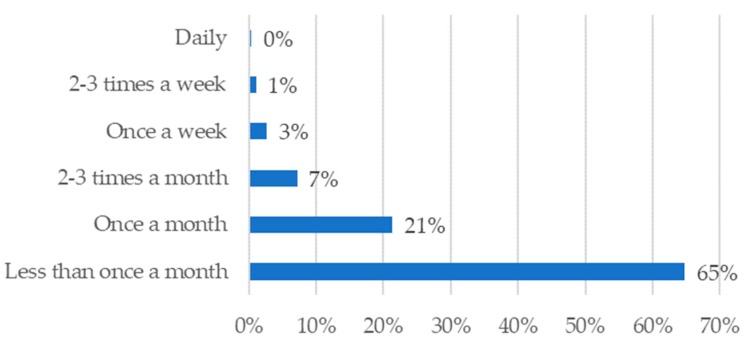
Frequency of insect incorporation.

Results indicate that incorporation occurs on an infrequent basis with 65% of respondents incorporating insects less than once a month during the school year (Figure 2). Lesson plans were used by 81% of teachers with nearly half of all teachers (49%) creating their own lesson plans. Lastly, 61% of teachers reported including live animals in their classrooms. Of those teachers using live animals, responses indicated that popular animal groups included insects (71%), non-insect arthropods (42%), fish (29%), reptiles (21%), amphibians (19%), annelids (19%), mollusks (14%), mammals (12%), cnidarians (9%), planarians (7%), birds (6%), and nematodes (2%).

**Figure 3:**
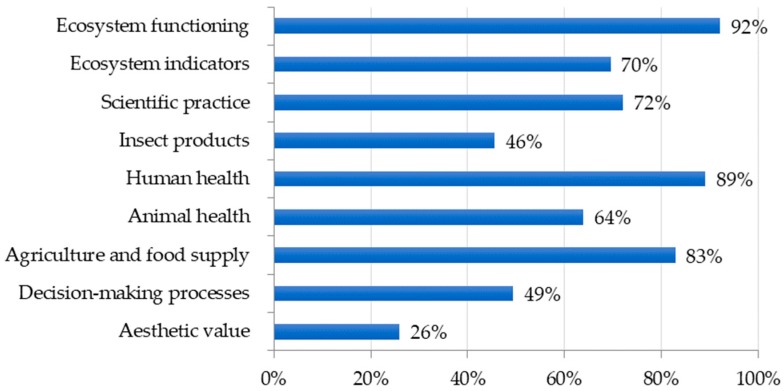
Entomology topics supported by insect incorporation.

In order to determine if students were being exposed to fundamental entomology topics, we used Pearson, Skinner, and Hoback [5] as a reference for what students should know and be able to do concerning entomology by the time they have left school. Insects were used to teach a variety of these entomology topics with ecosystem functioning, impacts on human health, and insects’ role in agriculture and our food supply being the most common topics covered. In contrast, aesthetic value of insects, the decision-making process of considering the costs and benefits of insect control, and value of insect products were introduced least often (Figure 3).

**Figure 4:**
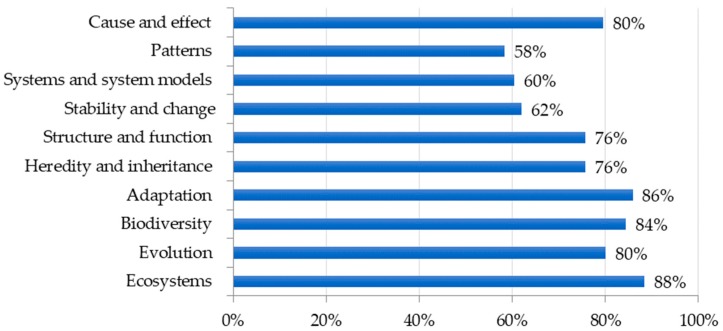
Life science concepts and core ideas supported by insect incorporation.

**Figure 5:**
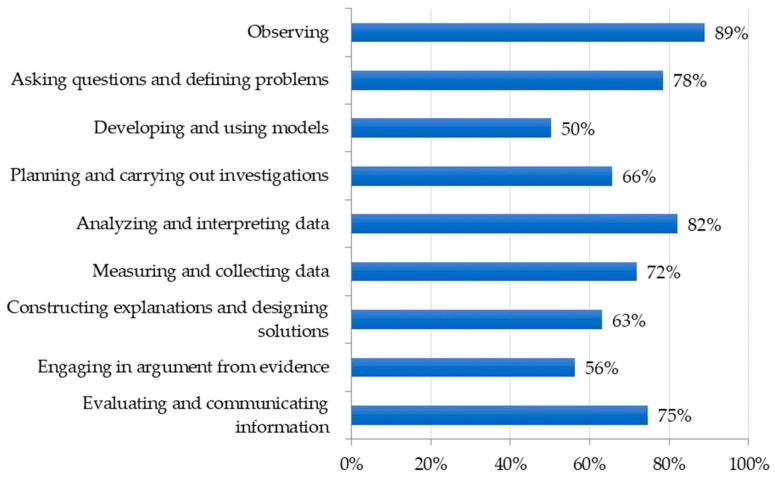
Science practices supported by insect incorporation.

Using the recently revised Next Generation Science Standards (NGSS) [10] as a guide, we made a list of concepts and disciplinary core ideas, which are commonly covered in life science instruction. While the NGSS were not adopted by all U.S. states at the time of this survey, the standards provide a framework of key science content and practices that we could reasonably expect teachers to use when selecting instructional resources. Teachers reported which concepts and disciplinary core ideas were most commonly supported by insect incorporation in their classroom (Figure 4). Similarly, the NGSS were used to identify a list of science practices which could be supported by insect incorporation. More than 75% of teachers indicated insects were used for observation, encouraging students to ask questions, analyzing or interpreting data, and evaluating and communicating information (Figure 5). Science practices such as developing and using models or engaging in argument from evidence were newly added to the revised standards. This may partially explain why insect incorporation is not yet encouraging these practices to a greater extent.

### 3.4. Barriers to Incorporation

**Figure 6:**
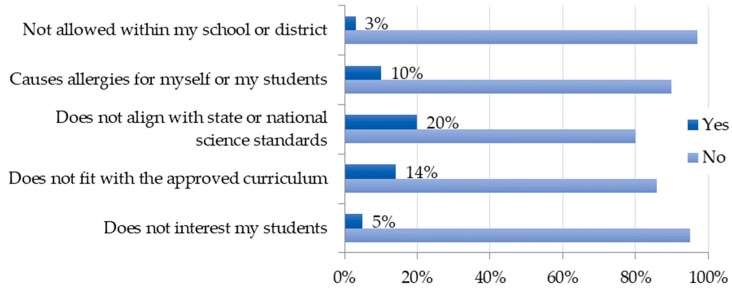
Barriers to insect incorporation.

**Figure 7:**
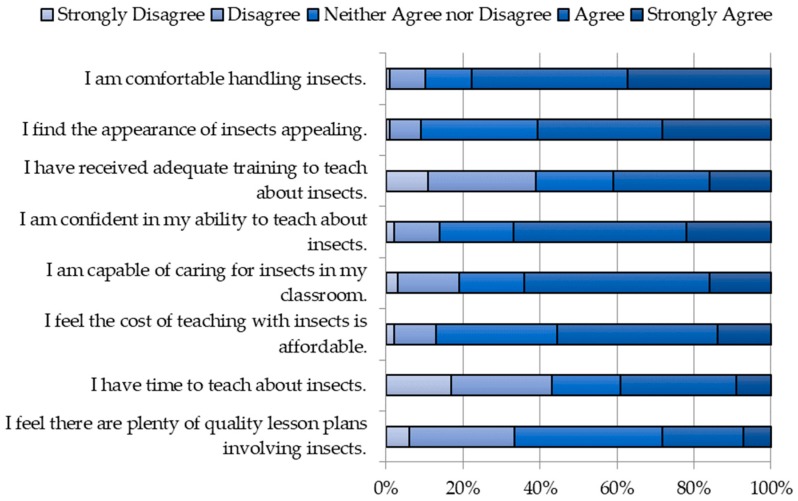
Teacher attitudes toward insect incorporation.

Teachers were asked to identify if any barriers existed hindering their incorporation of insects. The perceived lack of alignment with state or national science standards and lack of fit with a prescribed curriculum were the most common barriers reported (Figure 6). In addition, 40 teachers wrote in barriers including lack of time, prohibitive cost, and lack of facilities, knowledge, or ability to care for insects as potential barriers. These responses are not provided in Figure 6 because these teacher-generated responses were addressed within teacher attitudes in the following subsection (Figure 7).

### 3.5. Teacher Attitudes

In our survey sample, most teachers appeared to be comfortable with the physical appearance of insects (60%) and handling insects (77%). The most common concerns were lack of time to teach about insects (43%), lack of adequate training (39%), and lack of availability of quality lesson plans (33%) (Figure 7). Interestingly, despite 39% of teachers reporting a lack of adequate training, most teachers were confident in their ability to care for (64%) and teach about insects (67%).

### 3.6. Preferred Resources

When asked to rank six potential resources in terms of their usefulness to future insect incorporation, teachers ranked lesson plans aligned to state or national standards and professional development workshops teaching how to use insects to support inquiry as the top two most useful resources (Table 2).

## 4. Discussion

### 4.1. Findings

Findings from this study provide the entomology and science education communities with valuable insight into (1) how insect education is currently presented in a representative sample of U.S. secondary science classrooms, (2) potential obstacles that may hinder teachers from teaching about or with insects in formal secondary life science classrooms, and (3) opportunities to overcome these obstacles.

Our survey results describing how insect-related lessons are presented in secondary science classrooms may be best understood when viewed through the lens of standards-based science education reform in the United States. Contemporary U.S. education legislation requires that states adopt challenging academic content standards and evaluate student performance according to these standards [42]. To meet these directives, science teachers are tasked with selecting curriculum materials that support state-mandated science standards. Consequently, insect-related curriculum presented by teachers in our survey sample appear to be included in secondary science instruction largely based on perceived alignment with state standards. For example, while teachers reported incorporating an average of more than five different insect groups into science instruction, flies (order Diptera) were the most common insect group used. Prevalent incorporation of flies may be due, in part, to the emphasis placed on genetics as a core idea at the high school level in science standards [10] and the importance of *Drosophila melanogaster* (i.e., common fruit fly) as a popular model organism used in genetic studies. Further, teachers reported using insects to support teaching a large proportion of standards-related core ideas, concepts, and practices. In contrast, several topics deemed important by professional entomologists [5] such as recognizing aesthetic value of insects do not directly align with science standards and, perhaps as a result, were reported as being taught less. Finally, when asked to rank preferred resources, teachers overwhelmingly indicated the need for lesson plans aligned to standards and professional development focused on using insects to support inquiry, a central tenet of science education standards [9,10].

Previous studies of pre-service elementary teachers found patterns of teachers’ negative beliefs and attitudes towards invertebrates negatively influencing their reported willingness to use insects in future science instruction [43,44]. However, in identifying obstacles to incorporating insect education into high school science classrooms, we did not find evidence of negative teacher attitudes toward the appearance or handling of insects. Rather, our results suggest that teachers view a lack of time, training, and access to standards-aligned, insect-related teaching resources as leading challenges to greater insect incorporation. First, it is understandable that many teachers may struggle to find adequate instructional time for insect-related curriculum. As one teacher noted in the survey, “obstacles include teaching with insects in an already crowded curriculum by state mandate”. Next, in addition to a lack of time, teachers indicated the need for both professional development and standards-based lesson plans. If insects are to be used to support inquiry-based approaches, then professional development experiences will need to engage teachers in moving beyond the idea that insects simply provide a “wow” or “ick” factor to capture student attention. Through effective professional development experiences, teachers can integrate insects into science instruction in meaningful ways and engage students in authentic scientific inquiry practices. Lastly, it is possible teachers lack planning time needed to integrate new insect-related materials with their existing curriculum. While numerous standards-aligned entomology education resources exist, many teachers in our sample reported that they create their own lesson plans. While this may simply stem from teachers’ desire to write their own lessons, alternatively, it may indicate that teachers are unfamiliar with available materials, that available resources are not readily accessible, or that existing resources are not meeting the needs of secondary science teachers. Unless teachers are particularly committed to teaching with insects and developing their own lessons, this gap represents a potentially significant barrier to increased insect incorporation in secondary life science instruction.

To address the aforementioned teacher-identified obstacles, we recommend that invertebrate education and conservation organizations should consider partnering with curriculum developers and in-service teachers to develop education resources aligned to relevant state and national science standards. This collaboration of experts is critical in the development of scientifically accurate, classroom-centric, and standards-based educational materials. Through these collaborations, entomologists can help to incorporate entomological literacy concepts [5] into secondary classrooms. Expert-developed and standards-aligned curriculum would also save teachers valuable time in the planning process, thereby addressing teachers’ concerns about lack of time. In developing standards-aligned materials, it is important for those developers unfamiliar with standards-based science education reform to understand the transformation science education is currently undergoing. Since the 1990s, framework documents such as Science for All Americans from Project 2061 at AAAS [45] and the National Science Education Standards [9] have outlined what students should know and be able to do in order to become scientifically literate citizens. This effort has continued to evolve with the recently revised Next Generation Science Standards [10]. In alignment with science education reform recommendations, new entomology education resources should provide students with opportunities to engage in scientific practices in order to construct an understanding of core science ideas and concepts that are shared across the various science fields. Curriculum materials in insect education will need to reflect these fundamental changes in the current science education approach to fully meet the needs of today’s science educators. Next, to address issues of resource accessibility, a central database of teaching resources could be made available to teachers via an online web portal. To cultivate a community of practice around entomology education, this web portal could also include a message board or other means of online communication to support a network of teachers and entomology professionals. Finally, to address the need for teacher training, a variety of organizations and short-term grant-funded projects (e.g., Entomological Foundation’s STEM Bugs, Bugs in the Classroom, Bugging Out! Teaching with Insects, etc.) have previously been offered to support teachers’ professional development. In many cases, these programs provide positive, short-term experiences for teachers; however, development of sustained professional development programs are ideal to effect long-term, positive changes in secondary science teacher practice [46]. We suggest that entomology education organizations or university departments could partner with local school districts to provide teachers with professional development opportunities to expand their entomology expertise. These experiences could include developing long-term professional development opportunities or providing informal experiences focused on increasing teacher appreciation and attitude toward insects and other invertebrates.

### 4.2. Implications

This study provides a descriptive snapshot of insect incorporation in high school life science classrooms and insight into potential barriers preventing teachers from teaching about and with insects in formal secondary science settings. These insights are especially valuable as they come directly from in-service life science teachers who are in the best position to identify obstacles and provide their feedback on preferred resources to address identified barriers. With the knowledge gained from this survey, the entomology and science education communities are uniquely positioned to provide assistance directly to teachers and school districts most capable of enacting change.

### 4.3. Future Research Directions

Future qualitative research should be carried out to gather rich, descriptive data on the process that teachers experience when selecting and using entomology materials for use in their classrooms. A better understanding of this process could inform resource development and routes of delivery to best serve those secondary science teachers who would benefit from access to entomological instructional tools. In addition, understanding the perceived benefits of insect incorporation, such as increased interest in or improved attitude toward science or increased awareness of insects and their value, from both teacher and student perspectives could illuminate effective pathways toward entomological literacy. The qualitative approaches proposed would provide a more complete and detailed picture of insect incorporation at the secondary level.

## 5. Conclusions

Based on responses from a representative sample of U.S. secondary life science teachers, a wide variety of insects are incorporated into life science instruction, but that incorporation generally takes place less than once a month. Despite a trend of limited instructional time dedicated to insects, teachers report using insects to support a wide variety of science concepts associated with national science standards and student engagement in inquiry-based science practices. These findings suggest that the breadth and depth of insect incorporation in secondary science classrooms may be influenced by the degree to which teachers perceive insect-related resources as aligned with national or state science standards.

In addition to describing trends in entomology education in secondary life science classrooms, respondents identified key barriers to greater insect incorporation as lack of time, training, and standards-aligned resources. Teachers identified standards-aligned lesson plans and professional development workshops focused on using insects to support scientific inquiry as preferred resources for overcoming these barriers. Based on these findings, we recommend a careful and considered approach to development and dissemination of standards-aligned entomology education resources as well as creation and delivery of sustained professional development opportunities.

## Figures and Tables

**Table 1:** Comparison of U.S. teacher and school demographics to survey sample.

Demographic Type	Demographic Criteria	Demographic Characteristics	National Natural Science Teacher Population	Survey Sample	*n*	Difference
Teacher Demographics	Gender	Male	45%	38%	84	−7%
		Female	55%	62%	135	7%
	Age	Under 30	16%	6%	13	−10%
		30–39	30%	21%	46	−9%
		40–49	26%	29%	64	3%
		50–59	22%	33%	73	11%
		60 and over	7%	11%	23	4%
	Years Teaching	<3 years	10%	6%	11	−4%
		3–9	34%	15%	29	−19%
		10–20	36%	44%	86	8%
		>20 years	21%	35%	68	14%
	Degree Earned	<Bachelors	3%	0%	0	−3%
		Bachelors	36%	28%	61	−8%
		Masters	52%	64%	139	12%
		Doctorate	4%	7%	15	3%
		Education Specialist	6%	2%	4	−4%
School Demographics	Locale	City	26%	23%	48	−3%
		Suburb	27%	38%	81	9%
		Town	14%	14%	29	0%
		Rural	32%	26%	54	−6%
	Public/Private Designation	Public	75%	87%	181	+12%
		Private	25%	13%	27	−12%

**Table 2:** Teacher rankings of preferred resources.

Rank	Resource	Mean Rank ± SE
1	Lesson plans aligned with standards	2.61 ± 0.122
2	Professional development on using insects in inquiry	2.64 ± 0.111
3	Professional entomologists visiting the classroom	3.75 ± 0.123
4	Live insects available for check-out	3.78 ± 0.106
5	Insect collecting supplies available for check-out	4.00 ± 0.099
6	Guide on insect care	4.22 ± 0.104

## References

[1] Pedigo L.P., Rice M.E. (2009). Entomology and Pest Management.

[2] Triplehorn C.A., Johnson N.F., Borror D.J. (2005). Borror and DeLong’s Introduction to the Study of Insects.

[3] Shipley N.J., Bixler R.D. (2017). Beautiful bugs, bothersome bugs, and FUN bugs: Examining human interactions with insects and other arthropods. Anthrozoös.

[4] Kellert S.R. (1993). Values and perceptions of invertebrates. Conserv. Biol..

[5] Pearson G.A., Skinner K.M., Hoback W.W. (2007). Rearing the Masses: Defining Competencies for Entomological Literacy. Am. Entomol..

[6] Matthews R.W., Flage L.R., Matthews J.R. (1997). Insects as teaching tools in primary and secondary education. Annu. Rev. Entomol..

[7] Golick D.A., Heng-Moss T.M. (2013). Insects as educational tools: An online course teaching the use of insects as instructional tools. Am. Entomol..

[8] Golick D.A., Heng-Moss T.M., Ellis M.D. (2010). Using insects to promote science inquiry in elementary classrooms. NACTA J..

[9] National Research Council (1996). National Science Education Standards: Observe, Interact, Change, Learn.

[10] NGSS Lead States (2013). Next Generation Science Standards: For States, By States.

[11] Ashbrook P. (2007). Counting a culture of mealworms. Sci. Child..

[12] Hobbie A. (2000). Making Connections with insect royalty. Green Teach..

[13] Constible J., Lee R.E. (2006). Awesome Aggregations. Sci. Teach..

[14] Terry M. (2005). Art & Evolution. Sci. Teach..

[15] Gates D.M. (2005). Creating caddisfly cases. Sci. Scope.

[16] Damonte K. (2005). Exploring insect vision. Sci. Child..

[17] White H. (2009). Going Buggy. Sch. Art Educ. Mag. Teach..

[18] Travis H. (2003). Pheromone caterpillar trails: An easy lab exercise for the classroom. Am. Biol. Teach..

[19] Newell S.J. (1994). Occurrence of goldenrod galls: Study of insect ovipositing behavior. Am. Biol. Teach..

[20] Eason P.K., LaManna M.M. (2000). Testing folklore in the lab: Can common plants be used to repel insect pests?. Am. Biol. Teach..

[21] Bowen M.G. (2008). Investigating Crickets: Observing Animal Exploratory Behavior. Sci. Act. Classr. Proj. Curric. Ideas.

[22] Ashbrook P. (2007). Collards and Caterpillars. Sci. Child..

[23] Biggs D., Miller T., Hall D. (2006). There’s life in those dead logs!. Sci. Child..

[24] Gates D.M. (2002). Pond life magnified. Sci. Scope.

[25] Halverson K.L., Lankford D.M. (2009). Science galls me: What is a niche anyway?. Am. Biol. Teach..

[26] Hevel G. (2005). How do insects help the environment?. Sci. Child..

[27] Huss J., Baker C. (2010). The Farmer in the Lab. Sci. Child..

[28] Shepardson D.P. (2002). Bugs, butterflies, and spiders: Children’s understandings about insects. Int. J. Sci. Educ..

[29] Shepardson D.P. (1997). Of butterflies and beetles: First graders’ ways of seeing and talking about insect life cycles. J. Res. Sci. Teach..

[30] Killermann W. (1998). Research into biology teaching methods. J. Biol. Educ..

[31] Sammet R., Dreesmann D. (2017). What Do Secondary Students Really Learn during Investigations with Living Animals? Parameters for Effective Learning with Social Insects. J. Biol. Educ..

[32] Aschbacher P.R., Ing M., Tsai S.M. (2014). Is science me? Exploring middle school students’ STE-M career aspirations. J. Sci. Educ. Technol..

[33] Osborne J., Simon S., Collins S. (2003). Attitudes towards science: A review of the literature and its implications. Int. J. Sci. Educ..

[34] National Academy of Sciences, National Academy of Engineering (2005). Rising Above the Gathering Storm: Energizing and Employing America for a Brighter Economic Future.

[35] Barmby P., Kind P.M., Jones K. (2008). Examining changing attitudes in secondary school science. Int. J. Sci. Educ..

[36] Wang H.-H., Bhattacharya D., Evans E., Jirik P. (2017). Building Bee Houses: Designing and Constructing Solitary Bee Houses for Scientific Investigations. Sci. Scope.

[37] Golick D.A., Ellis M.D., Beecham B. (2006). Creating & Evaluating Artificial Domiciles for Bumble Bees. Am. Biol. Teach..

[38] Dillman D.A. (2009). Internet, Mail, and Mixed-Mode Surveys: The Tailored Design Method.

[39] Blank R., Langesen D., Petermann A. (2007). State Indicators of Science and Mathematics Education.

[40] U.S. Department of Education, National Center for Educational Statistics (2015). Table 209.50. Percentage of Public School Teachers of Grades 9 through 12, by Field of Main Teaching Assignment and Selected Demographic and Educational Characteristics: 2011–12.

[41] U.S. Department of Education, National Center for Educational Statistics (2015). Table 105.50. Number of Educational Institutions, by Level and Control of Institution: Selected Years, 1980–81 through 2013–14.

[42] (2015). Every Student Succeeds Act.

[43] Wagler R., Wagler A. (2011). Arthropods: Attitude and incorporation in preservice elementary teachers. Int. J. Environ. Sci. Educ..

[44] Wagler R., Wagler A. (2012). External insect morphology: A negative factor in attitudes toward insects and likelihood of incorporation in future science education settings. Int. J. Environ. Sci. Educ..

[45] American Association for the Advancement of Science (1989). Science for All Americans: A Project 2061 Report on Literacy Goals in Science, Mathematics, and Technology.

[46] Loucks-Horsley S. (2010). Designing Professional Development for Teachers of Science and Mathematics.

